# Experimental study to compare the strength of concrete with different amounts of polypropylene fibers at high temperatures

**DOI:** 10.1038/s41598-024-59084-6

**Published:** 2024-04-12

**Authors:** Yanhui Wang, Faezeh Nejati, S. A. Edalatpanah, Ramin Goudarzi Karim

**Affiliations:** 1 China Construction Third Engineering Bureau Group (Zhejiang) Co., Ltd., Zhejiang, China; 2 The Third Construction Co., Ltd., China Construction Third Engineering Bureau, Wuhan, China; 3 Department of Civil Engineering, Ayandegan Institute of Higher Education, Tonekabon, Iran; 4 Department of CIS, Stillman College, Tuscaloosa, AL USA

**Keywords:** Fiber concrete, High temperature, Fire, Polypropylene fibers, Civil engineering, Structural materials

## Abstract

It is widely known that adding fibers to concrete improves the properties of concrete, which has a brittle behavior. Although concrete has high compressive strength but poor tensile strength, this has led researchers to offer a variety of ways to deal with this weakness. The use of fibers is one of the methods used to enhance concrete behavior. Fire is one of the most important cases in structures; if the temperature is high or its duration is long, it will cause serious damage to the structure. The present study is an experimental study in which different concrete samples with different amounts of polypropylene fibers and different cement content are exposed once at a normal temperature of 25 °C and again at high temperatures, including 250 °C and 500 °C. The effect of temperature on the compressive and tensile strengths of concrete containing 0.5%, 1.5%, and 2% polypropylene fibres and with cement contents of 200 kg/m^3^, 260 kg/m^3^, and 320 kg/m^3^. The compressive and tensile strength was evaluated at curing 28 days of strength. The results showed a severe strength loss for all concretes after exposure to 500 °C. The relative compressive strengths of concretes containing PP fibers were higher than those of concretes without PP fibers. The tensile strength of concrete was more sensitive to high temperatures than the compressive strength. Based on the test results, it can be concluded that adding 2% PP fibers can significantly promote the residual mechanical properties of concrete during heating. The compressive strength at 25 °C with 2% PP fibres decreases by 43% with cement contents of 200 kg/m^3^ and 64% with cement contents of 260 kg/m^3^, and 37% with cement contents of 320 kg/m^3^, respectively. Also, the compressive strength at 500 °C with 2% PP fibres decreases by 61% with 200 kg/m^3^, 59% with 260 kg/m^3^, and 42% with cement contents of 320 kg/m^3^, respectively.

## Introduction

Fibres in different forms and materials are used to enhance the behaviour of concrete. Fiber-reinforced concrete is a type in which fibres are added to the concrete matrix constituents (water, cement, and aggregate) to improve performance. The fibres used in fibre-reinforced concrete can be randomly (discretely or continuously) distributed in the matrix and are mainly made of steel, aramid, glass, and polypropylene. According to research, using steel fibres to improve the tensile strength of concrete makes the concrete vulnerable to fire^1,2^. Global growth has led to an increased demand for construction materials. Researchers in the field of concrete have responded to this demand by developing various types of novel environmentally friendly materials^3^.

Won et al.^4^ studied the effect of polypropylene fibres on high-strength lightweight concrete. Based on their results, none of the specimens containing these fibres exhibited spalling or blow-up when exposed to high temperatures. Moreover, the results indicated that surface cracks in the samples decreased with increased polypropylene fibres.

Mazaheripour et al.^5^ investigated the impact of polypropylene fibres on the mechanical properties of fresh and hardened self-compacting lightweight concrete. Their results showed that adding polypropylene fibres equivalent to 0.3% of the concrete volume reduced the slump flow by 40% (from 720 to 430 mm). In addition, the polypropylene fibres did not affect the compressive strength and modulus of elasticity of the self-compacting lightweight concrete.

Caggiano et al.^6^ conducted compressive and bending tests on concrete specimens reinforced with polypropylene and steel fibres. Based on the results, the fibres had little effect on the compressive strength but caused an increase in elasticity. Unlike compressive strength, the overall shape of stress-crack opening-displacement graphs in the bending test highly depends on the type of fibres. Specimens with only polypropylene fibres showed excellent toughness after cracking for small crack opening ranges corresponding to the serviceability limit state.

Alsadey et al.^7^ examined the influence of polypropylene fibres on the mechanical properties of concrete. Three specimens with fibre contents of 1%, 1.5%, and 2%, as well as fibre-free samples, were tested to examine fibres' influence. According to the results, adding polypropylene fibres increased the compressive strength.

In another study, Alsadey et al.^8^ investigated the effect of polypropylene fibres on the mechanical characteristics of cement mortar. This study involved experiments on cement mortar reinforced with various amounts of polypropylene fibres, namely 0, 0.5%, 1%, and 1.5%. The flow table and compressive tests were performed on 28-day specimens. The results showed that an increase in the fibre content in cement mortar led to a significant increase in compressive strength.

Xu et al.^9^ evaluated polypropylene fibre-reinforced concrete’s stress–strain behaviour and damage mechanism under uniform and cyclic compression. For this purpose, 54 specimens with different volume percentages of fibre and different aspect ratios were tested. The acoustic emission technique was employed to analyze damage progress. Their results showed that adding polypropylene fibres increased toughness and ultimate strain and mitigated performance degradation to elastic stiffness and strength. Nevertheless, polypropylene fibres did not considerably affect plastic strain. Moreover, the effect of fibre volume fraction was more significant than the fibre aspect ratio on the cyclic behaviour of concrete.

Imansyah et al.^10^ investigated the effect of fibre and high-strength rebars on structural members. According to the results, using fibres increased the dissipated energy by about 27.5% compared to the specimen with regular concrete. In addition, the maximum strength was increased by 3–7% via fibres.

Aryanto et al.^11^ studied the behaviour of tensile concrete members with and without polypropylene fibres at different corrosion levels. The percentage of polypropylene fibres, which was taken to be 0.25%, 0.5%, 0.75%, and 1% by the total volume of concrete, was considered the primary variable in the concrete mixture. A comparison of the specimens with and without fibres and with the same corrosion percentage showed a reduction in the crack width in the samples with polypropylene fibres.

Zhenzhuan et al.^12^ conducted an analytical and experimental study of the explosion resistance of hybrid steel and ultrahigh-performance polypropylene-fiber-reinforced concrete under high temperatures. They compared the stress–strain curves after exposure of the specimens to temperatures of 200 °C, 400 °C, 600 °C, and 800 °C [unclear]. A good agreement was found between the analytical and experimental results.

Qiang et al.^13^ studied the durability and mechanical properties of rubber concrete containing basalt and polypropylene fibres. best high-temperature concrete performance was obtained with 1% and 1.5% volume fractions for the basalt and polypropylene fibres. Moreover, mixing basalt and polypropylene fibres was found to improve the performance of concrete at high temperatures.

In 2023, Fadi Althoey et al. investigated the experimental study on the properties of ultra-high-strength geopolymer concrete with polypropylene fibers and nano-silica At 750 °C elevated temperature, the samples' strength was reduced drastically, but at 250 °C, the modified samples showed good resistance to heat by retaining their compressive strength to some degree^14^.

Given the extensive body of research on the effect of fibres on tensile strength and researchers' concern about the low resistance of fibres against fire, In general, it can be concluded that the novelty of the research mentions that there is inconsistent information on the residual compressive strength of concrete with different percentage of PP fibers with different amount of cement dosage in the literature in high temperature. Furthermore, little information has been reported in the literature on the residual tensile strength of concretes containing different levels of PP fibers. This paper compares the compressive and tensile strength of concrete in two parts: (a) without PP fibers and (b) containing PP fibers at three different dosages.

## Materials used in the concrete

### Cement

Type-2 Tehran cement was used to prepare the concrete. Based on ASTM C150, this cement produces less heat than Type-1 Portland cement and is more resistant to sulfate corrosion^15^. The physical and chemical properties of the cement are shown in Tables 1 and 2.

**Table 1:** The chemical property of cement type-2.

No.	Component	Specification	Standard specification	Uncertainty	Standard method
1	SiO_2_	21.0 ± 0.5	> 20	0.14	1692
2	Al_2_O_3_	4.6 ± 0.15	< 6	0.02	
3	Fe_2_O_3_	3.9 ± 0.15	< 6	0.02	
4	CaO	62.5 ± 0.5	–	0.01	
5	MgO	2.9 ± 0.2	< 5	0.02	
6	SO_3_	2.0 ± 0.2	< 3	0.02	
7	Na_2_O	0.5 ± 0.05	–	–	1695
8	K_2_O	0.45 ± 0.05	< 3	–	
9	L.O.I	1.4 ± 0.3	< 0.75	0.03	1692
10	I.R	0.3 ± 0.1	–	0.02	
11	CI	–	–	–	–
12	Fr.CaO	–	–	–	–
13	C3S	54 ± 4	–	–	1692
14	C2S	23 ± 4	–	–	
15	C3A	5.6 ± 0.5	< 8	–	
16	C4AF	12 ± 1	–	–	–

**Table 2:** The physical properties of cement type-2.

No.	Component	Specification	Standard specification	Uncertainty	Standard method
1	Blaine (cm^2^/gr)	3200 ± 100	> 2800	79	390
2	Setting time (min)				
	Initial	200 ± 20	> 45	8	392
	Final	240 ± 20	< 360	52	
3	Compressive strength (kg/cm^2^)				
	1 day	–	–	–	393
	2 day	–	> 100	–	
	3 day	195 ± 20	> 175	18	
	7 day	310 ± 2	> 315	32	
	28 day	495 ± 20	< 0.8	55	
4	Autoclave expansion (%)				
	–	0.10 ± 0.02	–	0.14	391

### Aggregate

**Figure 1:**
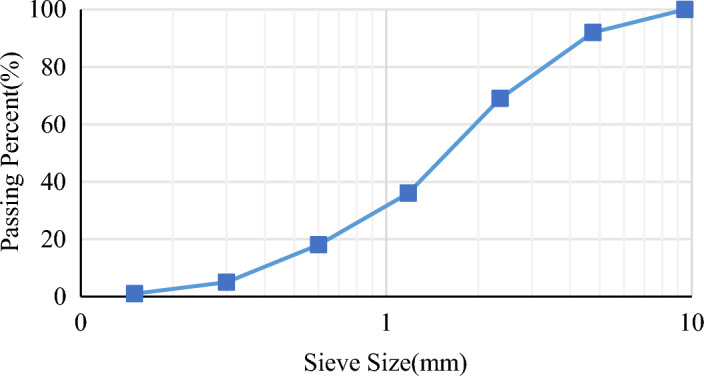
Gradation curve.

The properties of cement, including its compressive strength, depend on the characteristics of the aggregates used in it. Thus, good-quality aggregate must be used to prepare the cement. To have dense concrete, one must use aggregates with a wide range of sizes. As such, the smaller aggregates fill in the gaps between the larger aggregates, resulting in stronger concrete^16^. The aggregate used in this research consisted of clinker and sand. Clinker is a type of lightweight aggregate with through pores. Hence, it must reach the appropriate moisture level before mixing to not absorb water from the concrete during mixing. The clinker used in this research had grains of 0–10 mm. The sand gradation is shown in Fig. 1.

### Superplasticizer

A polycarboxylate superplasticizer was used to prepare the concrete in this research. The amount of superplasticizer commonly used in the concrete varies from 0.2 to 0.8% of the cement's weight depending on the aggregate's type and size, cement content, water-to-cement ratio, air temperature, and preparation method^17^. In this research, the amount of polycarboxylate used in the mix design was 0.8% of the weight of cement. This superplasticizer reduces the water-to-cement ratio and improves cement dispersion. The reduction in the water-to-cement ratio decreases the capillary pores in the concrete and increases its strength. Superplasticizers prevent the attraction of cement particles with their electrostatic repulsion. As a result, they reduce the need for water in the cement and improve its performance.

### Water

The amount and quality of water used in preparing concrete significantly affect its quality. This amount is about 15–25% of the volume of concrete^18^. This study used tap water in Tehran to prepare the concrete with a water-to-cement ratio of 0.45.

### Polypropylene fibers

**Figure 2:**
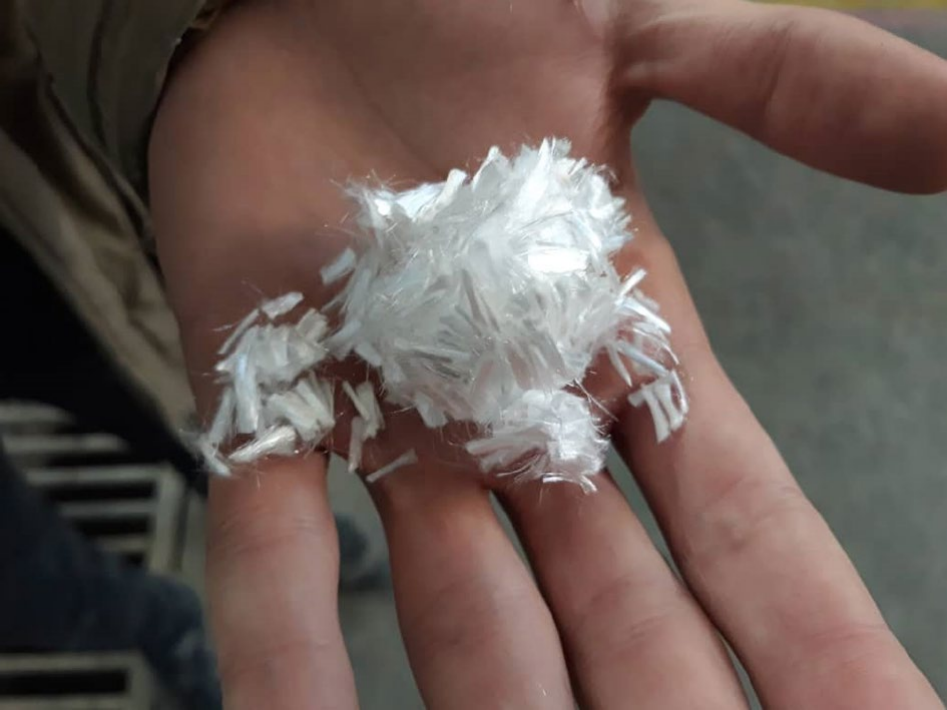
Appearance of polypropylene fibers.

Polypropylene fibres have high tensile strength and a low modulus of elasticity. Using these fibres in concrete increases its elasticity and impact resistance and prevents it from blowing up at high temperatures. The post-cracking behaviour of fibre-reinforced concrete depends on the fibre-matrix bond. If these two are mixed well, and the bond strength can withstand the forces created in the fibres, the failure will be fibre breakage; otherwise, it will be fibre pull-out^19,20^. This work used 0.5%, 1.5%, and 2% fibers. Table 3 and Fig. 2 present the specifications and appearance of the polypropylene fibres used in this research.

**Table 3:** Specifications of polypropylene fibers.

Fiber type	Melting point (°C)	Tensile strength (MPa)	Length (mm)	Diameter (µm)
Polypropylene	165	350	6	24

## Mix design

This research used nine different mix designs to prepare the specimens to study the effect of fibre percentage and cement content on concrete's compressive and tensile strength at temperatures of 25 °C, 250 °C, and 500 °C. These mix designs included fibre contents of 0.5%, 1.5%, and 2% and cement contents of 200 kg/m^3^, 260 kg/m^3^, and 320 kg/m^3^. Mixing designs No. 1, 2, and 3 is related to the 200 kg/m3 cement content and fibers with percentages of 0.5, 1.5, and 2%. Mixing designs No. 4, 5, and 6 is related to the 260 kg/m3 cement content and fibers with percentages of 0.5, 1.5, and 2%. Mixing designs No. 7, 8, and 9 is related to the cement content of 320 kg/m^3^ and fibers with percentages of 0.5, 1.5, and 2%. Moreover, the ratios of water and superplasticizer to cement were 0.45 and 0.80%, respectively, in all the mix designs. Furthermore, sand contents of 321 kg/m^3^, 304 kg/m^3^, and 286 kg/m^3^ and clinker contents of 508 kg/m^3^, 480 kg/m^3^, and 452 kg/m^3^ were used. Table 4 presents the mix designs utilized to prepare concrete in this research.

**Table 4:** Mix designs used for preparing concrete.

Materials	Mix design 1	Mix design 2	Mix design 3	Mix design 4	Mix design 5	Mix design 6	Mix design 7	Mix design 8	Mix design 9
Sand (kg/m^3^)	321	304	286	321	304	286	321	304	286
Clinker (kg/m^3^)	508	480	452	508	480	452	508	480	452
Water (kg/m^3^)	90	117	144	90	117	144	90	117	144
Cement (kg/m^3^)	200	260	320	200	260	320	200	260	320
Polypropylene fibers (kg/m^3^)	4.55	4.55	4.55	4.55	4.55	4.55	4.55	4.55	4.55
Superplasticizer-to-cement ratio (%)	0.80	0.80	0.80	0.80	0.80	0.80	0.80	0.80	0.80

A furnace with dimensions of 30 × 30 × 40 cm and a maximum temperature of 1500 °C was prepared to subject the specimens to high temperatures. The furnace used an element to generate heat at a rate of 4.17 °C/min.

## Experimental tests

Cubic metal molds with dimensions of 10 × 10 × 10 cm and cylindrical 15 × 30 cm were used to make compression and tension samples, respectively. After compacting and polishing the concrete, the samples were left in the laboratory environment for 24 h for the concrete to set. The samples were removed from the mold and placed inside the water for 28 days for the curing operation. After the treatment, the samples were placed in the laboratory environment to dry. Then, the samples were placed inside the furnace at 250 and 500 degrees Celsius.

The furnace temperature takes 1 and 2 h to reach 250 and 500 °C, respectively. After the heated furnace reached the desired temperature, the samples were kept at that temperature for 3 h. The samples were kept open in the furnace for 24 h to reach the ambient temperature, and compressive and tensile strength tests were performed on them. To expose the samples to high heat, a handmade oven with dimensions of 30 × 30 × 40 cm with the capacity to produce temperatures up to 1500 degrees Celsius was used. The element is used to generate heat inside the furnace, and the rate of heat application is equal to 17.4 degrees Celsius per minute.

A UTM machine with a capacity of 100 tons was used to perform the compressive and tensile tests. The loading speed applied to the samples is 1 mm/min.

Compressive strength test is the most common and most important test on concrete. In this research, the compressive strength test based on BS 1881-124:2015 Testing concrete was done on cubic samples with dimensions of 10 × 10 × 10 cm.

In this test, the samples were placed between the two upper and lower jaws of the concrete breaker jack so that the concreting direction was perpendicular to the displacement direction. Nine cubic samples with different mixing designs were subjected to compression tests for each tested temperature.

In this research, the Brazilian method of splitting was used based on the ASTM C496 standard to determine the tensile strength of concrete. This test was performed on cylindrical samples with 15 × 30 cm dimensions.

## Result and discussion

### Effect of temperature on compressive and tensile strength

**Figure 3:**
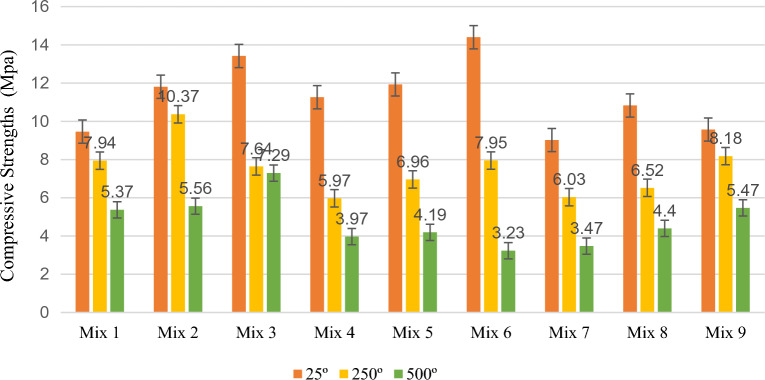
A comparison between the compressive strengths of the specimens at various temperatures and mix designs.

**Figure 4:**
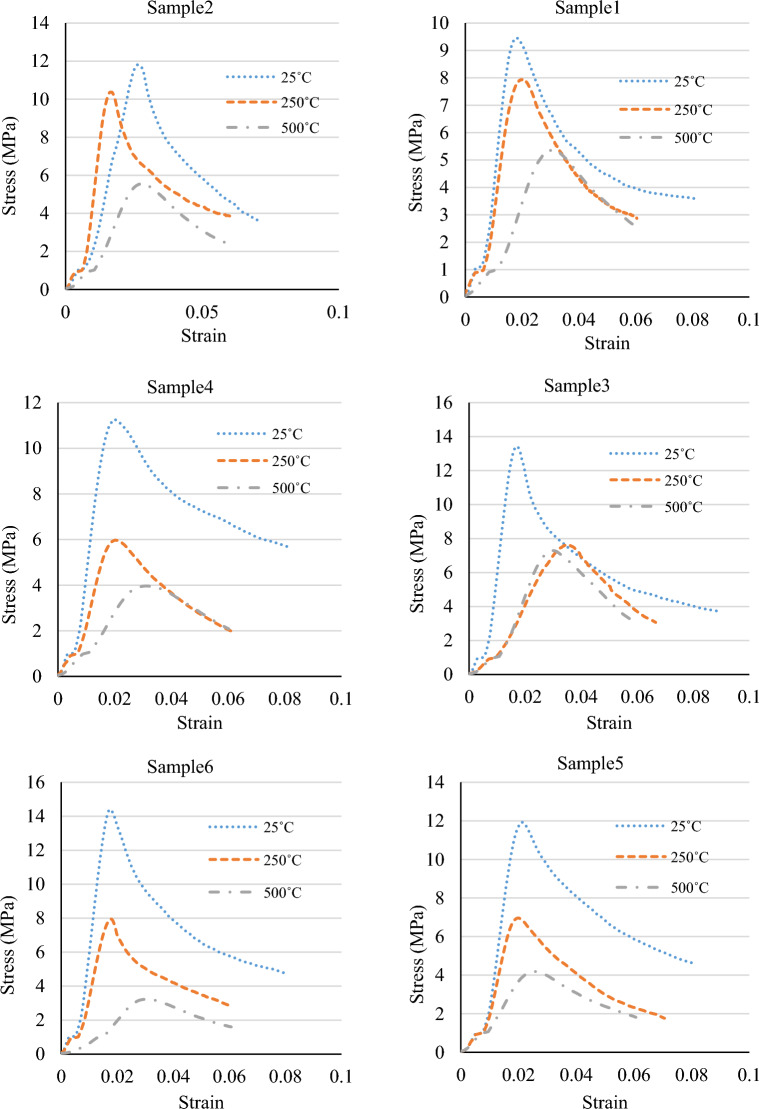

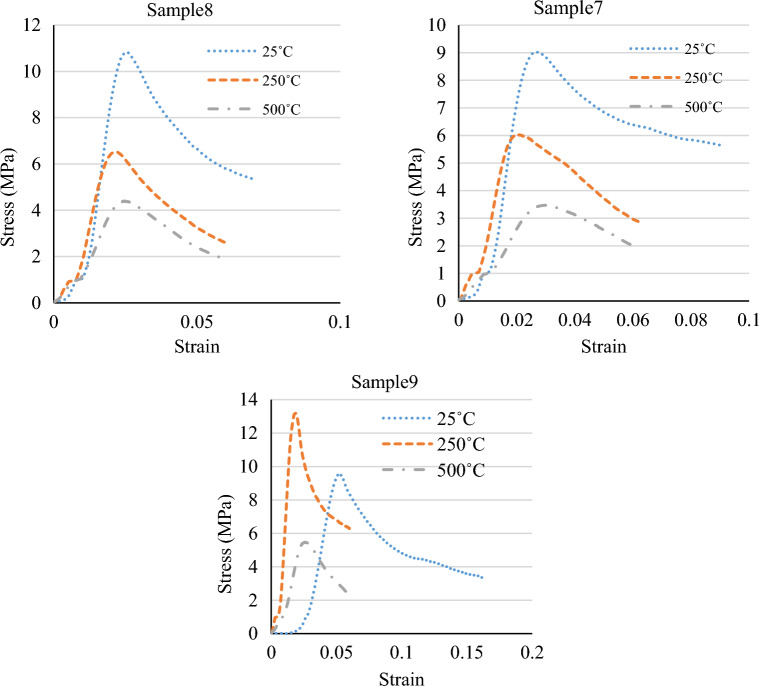
The stress–strain graphs of the specimens at various temperatures and mix designs.

Figures 3 and 4 display the compressive strength and stress–strain graphs from the compression tests at 25 °C, 250 °C, and 500 °C. Table 5 shows the compressive strength of the specimen at temperatures of 25 °C , 250 °C, and 500 °C.

**Table 5:** The compressive strength of the specimen at temperatures of 25 °C, 250 °C, 500 °C.

Specimen temperature	Fiber content of 0.5%			Fiber content of 1.5%			Fiber content of 2%		
	Cement content of 320 (kg/m^3^)	Cement content of 260 (kg/m^3^)	Cement content of 200 (kg/m^3^)	Cement content of 320 (kg/m^3^)	Cement content of 260 (kg/m^3^)	Cement content of 200 (kg/m^3^)	Cement content of 320 (kg/m^3^)	Cement content of 260 (kg/m^3^)	Cement content of 200 (kg/m^3^)
25 °C	9.02	11.26	9.46	10.83	11.93	11.81	9.57	14.4	13.42
250 °C	6.03	5.97	7.94	6.52	6.96	10.37	8.18	7.95	7.64
500 °C	3.47	3.97	5.37	4.4	4.19	5.56	5.47	3.23	7.29

The compressive strength of specimens with 0.5% fibre at temperatures of 25 °C, 250 °C, and 500 °C was found to be, respectively, 9.46 MPa, 7.94 MPa, and 5.37 MPa for a cement content of 200 kg/m^3^, 11.81 MPa, 10.37 MPa, and 5.56 MPa for a cement content of 260 kg/m^3^; and 13.42 MPa, 7.64 MPa, and 7.29 MPa for a cement content of 320 kg/m^3^. The results indicated that the compressive strength decreased with a rise in temperature at various cement contents in the specimens with 0.5% fibre. The most significant decrease in compressive strength relative to the sample at 25 °C occurred at 500 °C with a cement content of 260 kg/m^3^ and was equal to 53%.

The compressive strength of specimens with 1.5% fibre at temperatures of 25 °C, 250 °C, and 500 °C was found to be, respectively, 11.26 MPa, 5.97 MPa, and 3.97 MPa for a cement content of 200 kg/m3, 11.93 MPa, 6.96 MPa, and 4.19 MPa for a cement content of 260 kg/m3; and 14.40 MPa, 7.95 MPa, and 3.23 MPa for a cement content of 320 kg/m3. The results showed that the compressive strength decreased with the increased temperature at different cement contents in the specimens with 1.5% fibre. The most significant decrease in compressive strength relative to the sample at 25 °C occurred at 500 °C with a cement content of 260 kg/m3 and was equal to 78%.

Moreover, the compressive strength of specimens with 2% fibre at temperatures of 25 °C, 250 °C, and 500 °C was obtained to be, respectively, 9.02 MPa, 6.03 MPa, and 3.47 MPa for a cement content of 200 kg/m3, 10.83 MPa, 6.52 MPa, and 4.40 MPa for a cement content of 260 kg/m3; and 9.57 MPa, 8.18 MPa, and 5.47 MPa for a cement content of 320 kg/m3. Based on the results, the compressive strength decreased with an increase in temperature at cement contents of 200 kg/m3 and 260 kg/m3 in the specimens with 2% fibre. Also, the largest drop in compressive strength relative to the specimen at 25 °C occurred in the sample at 500 °C with a cement content of 200 kg/m3 and equaled 62%.

According to the results, the compressive strength was reduced by increased temperature in all the specimens. It has been well known that the pore vapor pressure depends on the porosity of concrete. Since the PP fibers melt before reaching 250 °C, the porosity of the concrete is increased, and more escape routes were added to reduce the water vapor pressure. Furthermore, the disappearance of PP fibers may reduce the results of thermal incompatibility between aggregates and paste due to the provision of more free space and the fact that they act as thermal shock absorbers. After exposure to 500 °C, several researchers reported the relative residual compressive strengths dropped very sharply^21^.

Ignoring the sample with a cement content of 320 kg/m^3^ and fibre content of 2%, the compressive strength decreased on average by 35% at a temperature of 250 °C and by 57% at a temperature of 500 °C. In addition, the results showed that Mix Design 3 exhibited the highest compressive strength at a temperature of 500 °C. This was due to the mix design's lower fibre content and higher cement content than the others. High temperatures cause evaporation of the fibres and the creation of interstices in the concrete. As a result, a smaller fibre content means fewer interstices, which leads to a higher concrete strength. Furthermore, the cohesiveness of cement causes an increase in the cement content to increase the strength of concrete. Such response is most likely due to reduced porosity in high-strength concrete with smaller and less interconnected pores, favoring vapor pressure build-up and reduced thermal diffusivity reported in several research studies^21^.

Also, Table 6 presents the percentage difference of the compressive strengths of the specimens at temperatures of 250 °C and 500 °C from those of the samples at 25 °C.

**Table 6:** Percentage difference in compressive strength relative to the specimen at a temperature of 25 °C.

Specimen temperature	Fiber content of 0.5%			Fiber content of 1.5%			Fiber content of 2%		
	Cement content of 320 (kg/m^3^)	Cement content of 260 (kg/m^3^)	Cement content of 200 (kg/m^3^)	Cement content of 320 (kg/m^3^)	Cement content of 260 (kg/m^3^)	Cement content of 200 (kg/m^3^)	Cement content of 320 (kg/m^3^)	Cement content of 260 (kg/m^3^)	Cement content of 200 (kg/m^3^)
250 °C	− 43	− 12	− 16	− 45	− 42	− 47	− 14	− 40	− 33
500 °C	− 46	− 53	− 43	− 78	− 65	− 65	− 43	− 59	− 62

**Figure 5:**
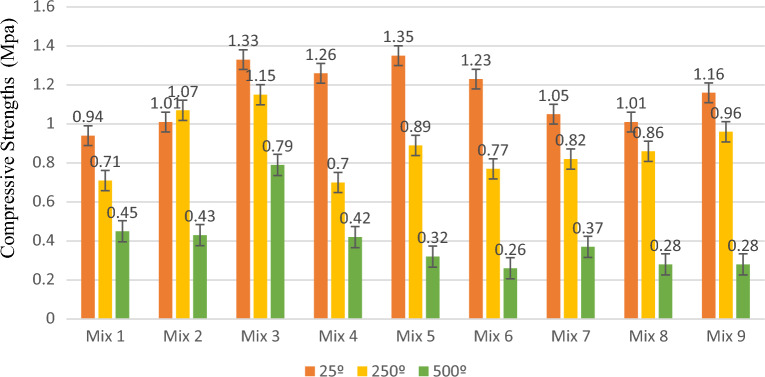
A comparison between the compressive strengths of the specimens at various temperatures and mix designs.

**Figure 6:**
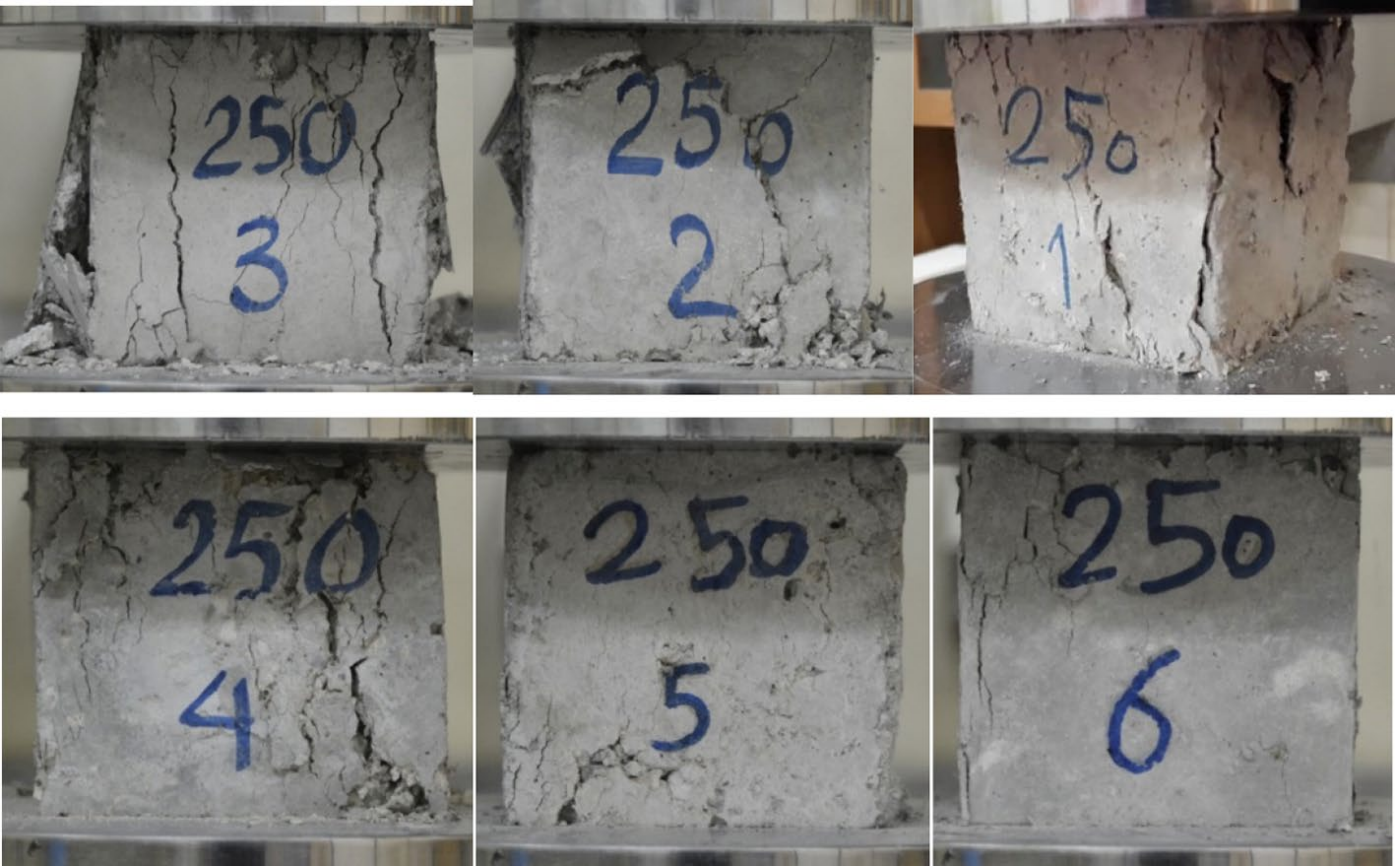
Cracks and brittleness of samples exposed to 250˚C temperature under pressure test.

**Figure 7:**
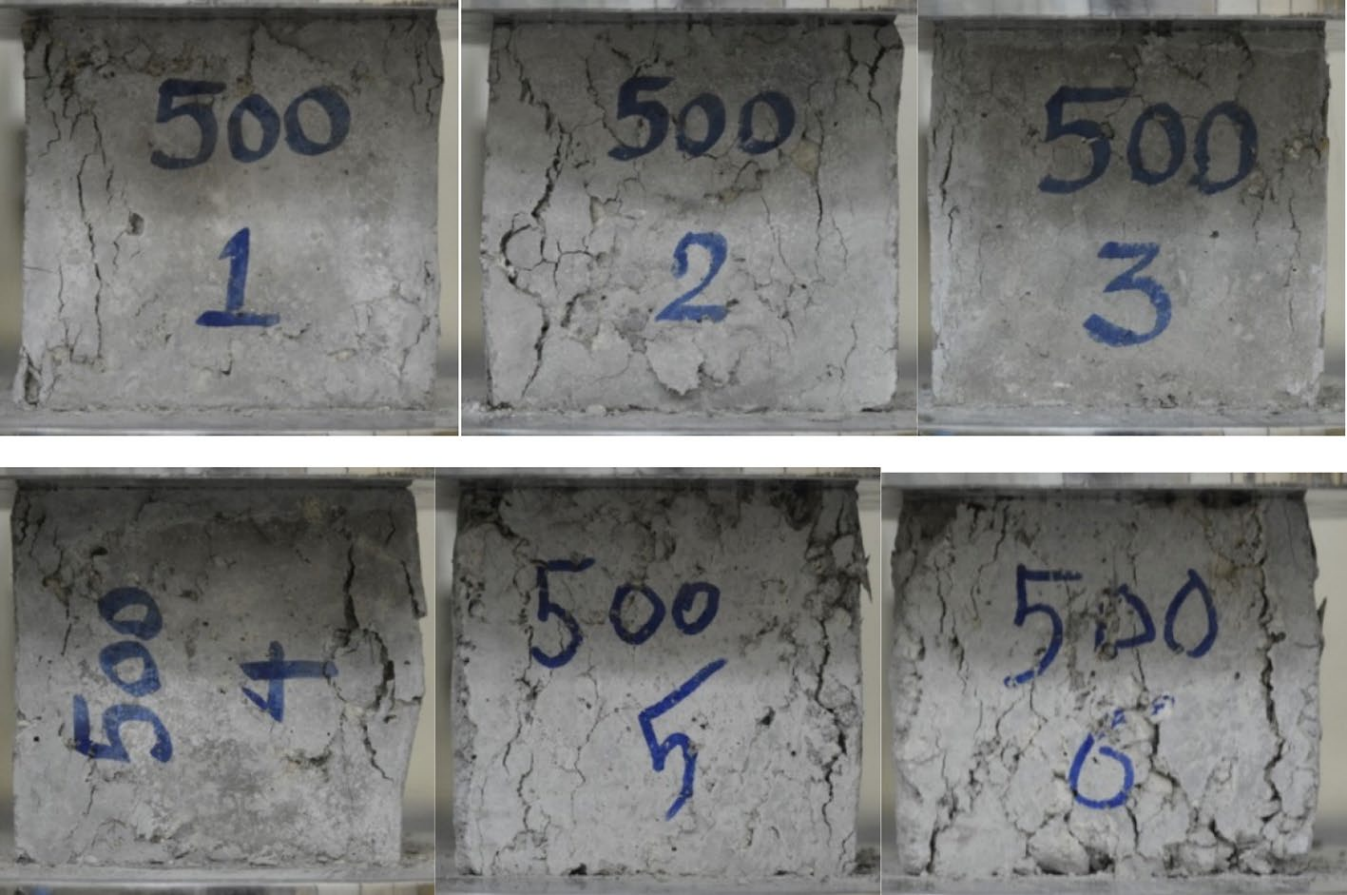
Cracks and brittleness of samples exposed to 500˚C temperature under pressure test.

Figure 5 depicts the tensile strength and stress–strain graphs from the tensile tests at 25 °C, 250 °C, and 500 °C. Also, Figs. 6 and 7 show the sample in high temperatures. Moreover, Table 7 presents the percentage difference of the tensile strengths of the specimens at temperatures of 250 °C and 500 °C from those of the samples at 25 °C.

**Table 7:** Percentage difference in tensile strength relative to the specimen at a temperature of 25 °C.

Fiber content of 2%			Fiber content of 1.5%			Fiber content of 0.5%			Specimen temperature
Cement content of 320 (kg/m^3^)	Cement content of 260 (kg/m^3^)	Cement content of 200 (kg/m^3^)	Cement content of 320 (kg/m^3^)	Cement content of 260 (kg/m^3^)	Cement content of 200 (kg/m^3^)	Cement content of 320 (kg/m^3^)	Cement content of 260 (kg/m^3^)	Cement content of 200 (kg/m^3^)	
− 17	− 15	− 22	− 37	− 34	− 44	− 14	6	− 24	250 °C
− 76	− 72	− 65	− 79	− 76	− 67	− 41	− 57	− 52	500 °C

The tensile strength of specimens with 0.5% fibre at temperatures of 25 °C, 250 °C, and 500 °C was obtained to be, respectively, 0.94 MPa, 0.71 MPa, and 0.45 MPa for a cement content of 200 kg/m^3^, 1.01 MPa, 1.07 MPa, and 0.43 MPa for a cement content of 260 kg/m^3^; and 1.33 MPa, 1.15 MPa, and 0.79 MPa for a cement content of 320 kg/m^3^. Based on the results, the tensile strength decreased with an increase in temperature at cement contents of 200 kg/m^3^ and 320 kg/m^3^ in the specimens with 0.5% fibre. The specimen at 500 °C with a cement content of 260 kg/m^3^ exhibited the most significant decrease in tensile strength relative to the sample at 25 °C, which was equal to 57%.

The tensile strength of specimens with 1.5% fibre at temperatures of 25 °C, 250 °C, and 500 °C was determined to be, respectively, 1.26 MPa, 0.70 MPa, and 0.42 MPa for a cement content of 200 kg/m^3^, 1.35 MPa, 0.89 MPa, and 0.32 MPa for a cement content of 260 kg/m^3^; and 1.23 MPa, 0.77 MPa, and 0.26 MPa for a cement content of 320 kg/m^3^. According to the results, the tensile strength decreased with a rise in temperature at different cement contents in the specimens with a fibre content of 1.5%. The sample at 500 °C with a cement content of 320 kg/m^3^ experienced the most significant decrease in tensile strength relative to the specimen at 25 °C, which was equal to 79%.

The tensile strength of specimens with 2% fibre at temperatures of 25 °C, 250 °C, and 500 °C was obtained to be, respectively, 1.50 MPa, 0.82 MPa, and 0.37 MPa for a cement content of 200 kg/m^3^; 1.01 MPa, 0.86 MPa, and 0.28 MPa for a cement content of 260 kg/m^3^; and 1.16 MPa, 0.96 MPa, and 0.28 MPa for a cement content of 320 kg/m^3^. Based on the results, the tensile strength decreased with a rise in temperature at different cement contents in the specimens with a fibre content of 2%. The sample at 500 °C with a cement content of 320 kg/m^3^ exhibited the most significant decrease in tensile strength relative to the specimen at 25 °C, which was equal to 76%.

The results also indicated that tensile strength decreased with increased temperature, similar to compressive strength. The higher reductions in the tensile strengths of fiber concretes at 500 °C when compared to 250 °C may be attributed to the coarsening effect of high temperature on the pore size distribution reported in several researches^22–24^ as well as the higher porosity of fiber concretes at this temperature. Ignoring the specimen with a cement content of 260 kg/m^3^ and a fiber content of 1.5%, the tensile strength decreased on average by 26% at 250 °C and by 65% at a temperature of 500 °C. In addition, the most considerable reduction in strength occurred in the specimens with a fibre content of 1.5%. This can be because fewer pores were created in the samples with 0.5% fibre due to the small amount of evaporated fibre and some remaining fibres in the specimens with 2% fibre due to the insufficient time for the evaporation of all the fibres. In addition, the results showed that Mix Design 3 experienced the highest tensile strength at a temperature of 500 °C. This was due to the mix design's lower fibre content and higher cement content than the others. High temperatures lead to fibre evaporation and pore creation in the concrete. Consequently, a smaller fibre content means fewer pores, which leads to a higher concrete strength. In addition, due to the cohesiveness of cement, a rise in the cement content increases the strength of concrete.

### Effect of fiber content on compressive and tensile behavior

**Figure 8:**
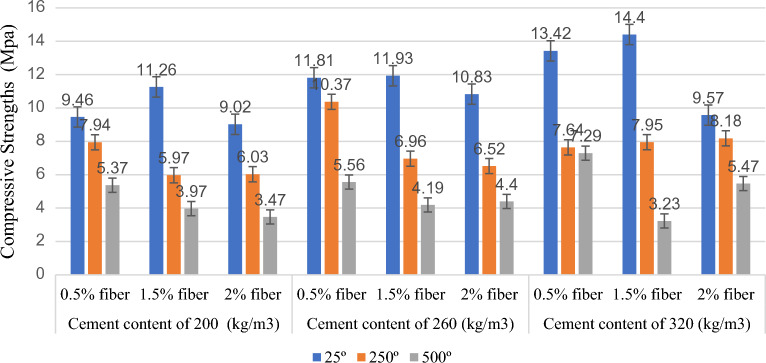
A comparison between the compressive strengths of the specimens at various temperatures and mix designs.

**Figure 9:**
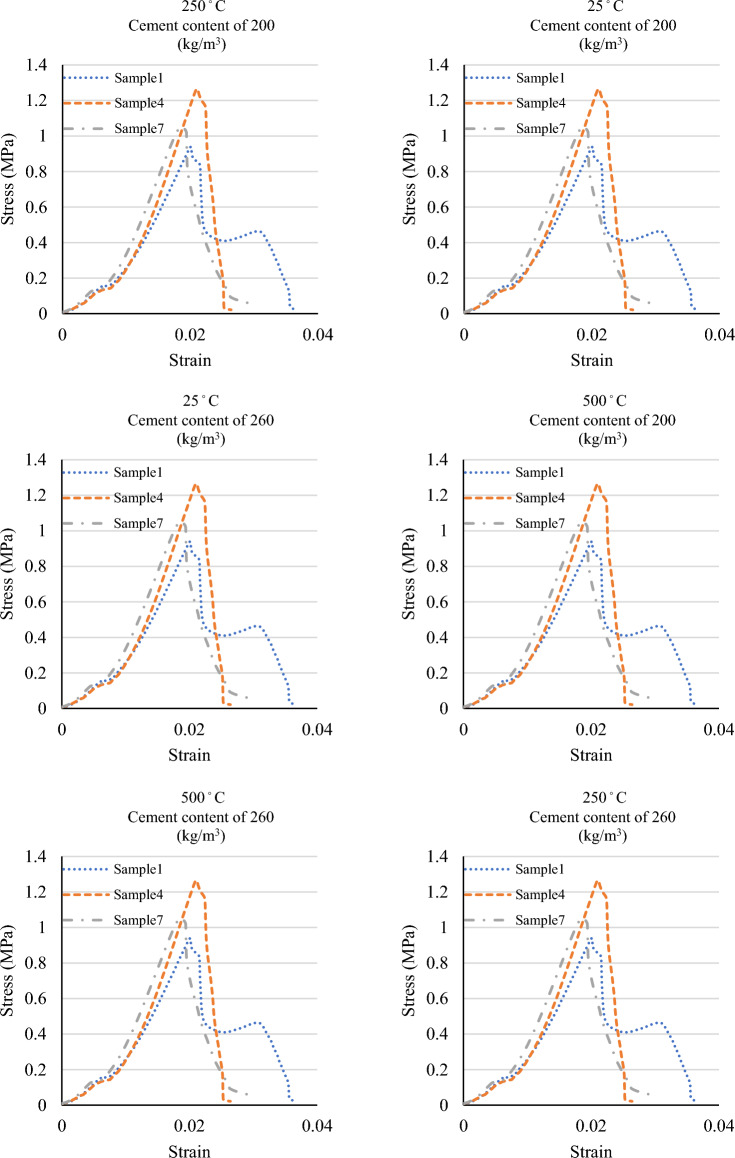

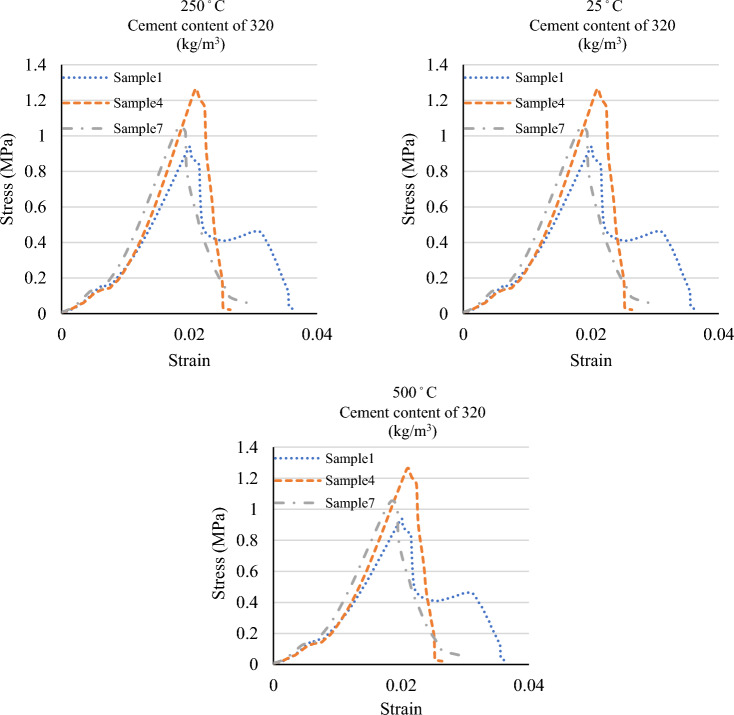
A comparison between the stress–strain graphs of the specimens at various temperatures and mix designs.

Figures 8 and 9 display the compressive strength and stress–strain curves obtained from the compression tests at 0.5%, 1.5%, and 2% fibre contents. The compressive strength was evaluated at curing of 28 days of strength. Also, Table 8 presents the percentage difference of the compressive strengths of the specimens with 1.5% and 2% fibre from the compressive strengths of those with 0.5% fibre.

**Table 8:** Percentage difference in the compressive strength relative to the specimen with 0.5% fiber.

Fiber content (%)	Temperature of 25 °C			Temperature of 250 °C			Temperature of 500 °C		
	Cement content of 320 (kg/m^3^)	Cement content of 260 (kg/m^3^)	Cement content of 200 (kg/m^3^)	Cement content of 320 (kg/m^3^)	Cement content of 260 (kg/m^3^)	Cement content of 200 (kg/m^3^)	Cement content of 320 (kg/m^3^)	Cement content of 260 (kg/m^3^)	Cement content of 200 (kg/m^3^)
1.5	7	1	19	4	− 33	− 25	− 56	− 25	− 26
2	− 29	− 8	− 5	73	− 37	− 24	− 25	− 21	− 35

The compressive strength of specimens at 25 °C with a fibre content of 0.5%, 1.5%, and 2% was obtained to be, respectively, 9.46 MPa, 11.26 MPa, and 9.02 MPa for a cement content of 200 kg/m3; 11.81 MPa, 11.93 MPa, and 10.83 MPa for a cement content of 260 kg/m^3^; and 13.42 MPa, 14.40 MPa, and 9.57 MPa for a cement content of 320 kg/m^3^. In the specimens at 25 °C and with various cement contents, the highest compressive strength occurred in those with 1.5% fibre. Moreover, the compressive strength of the samples with 2% fibre was smaller than that of the specimens with 0.5% fibre.

The compressive strength of specimens at 250 °C with a fibre content of 0.5%, 1.5%, and 2% were found to be, respectively, 7.94 MPa, 5.97 MPa, and 6.03 MPa for a cement content of 200 kg/m^3^; 10.37 MPa, 6.96 MPa, and 6.52 MPa for a cement content of 260 kg/m^3^; and 7.64 MPa, 7.95 MPa, and 18.93 MPa for a cement content of 320 kg/m^3^. At a temperature of 250 °C, an increase in the fibre content decreased the compressive strength in the specimens with a cement content of 200 kg/m^3^ and 260 kg/m^3^ but increased the compressive strength in those with a cement content of 320 kg/m^3^.

The compressive strength of specimens at 500 °C with a fibre content of 0.5%, 1.5%, and 2% was determined to be, respectively, 5.37 MPa, 3.97 MPa, and 3.47 MPa for a cement content of 200 kg/m^3^; 5.56 MPa, 4.19 MPa, and 4.40 MPa for a cement content of 260 kg/m^3^; and 7.29 MPa, 3.23 MPa, and 5.47 MPa for a cement content of 320 kg/m^3^. An increase in fiber percentage in the specimens at 500 °C and with various cement contents reduced compressive strength. Moreover, the compressive strength of the sample with 1.5% fibre was more extensive than that of the sample with 2% fibre at 260 kg/m^3^ and 320 kg/m^3^ cement contents.

The results indicated that an increase in the fibre content of the specimens subjected to temperatures of 250 °C and 500 °C led to a decrease in compressive strength. The fibres melt and evaporate at high temperatures, leaving pores in the concrete. Hence, an increase in the percentage of fibres increases the concrete pores and further reduces its strength. Moreover, the results showed that, at a temperature of 25 °C, the compressive strength increased for specimens with a fibre content of 1.5% and decreased for those with a fibre content of 2%.

**Figure 10:**
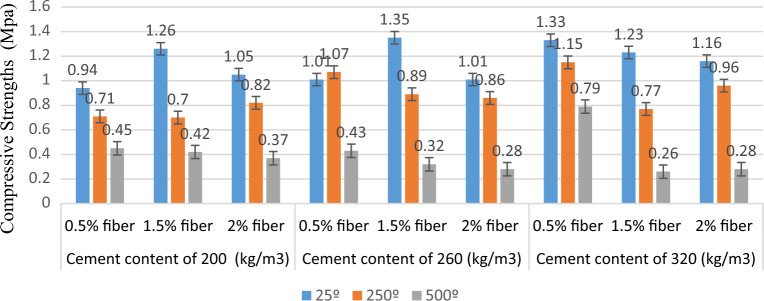
A comparison between the tensile strength of the specimens at various temperatures and mix designs.

**Figure 11:**
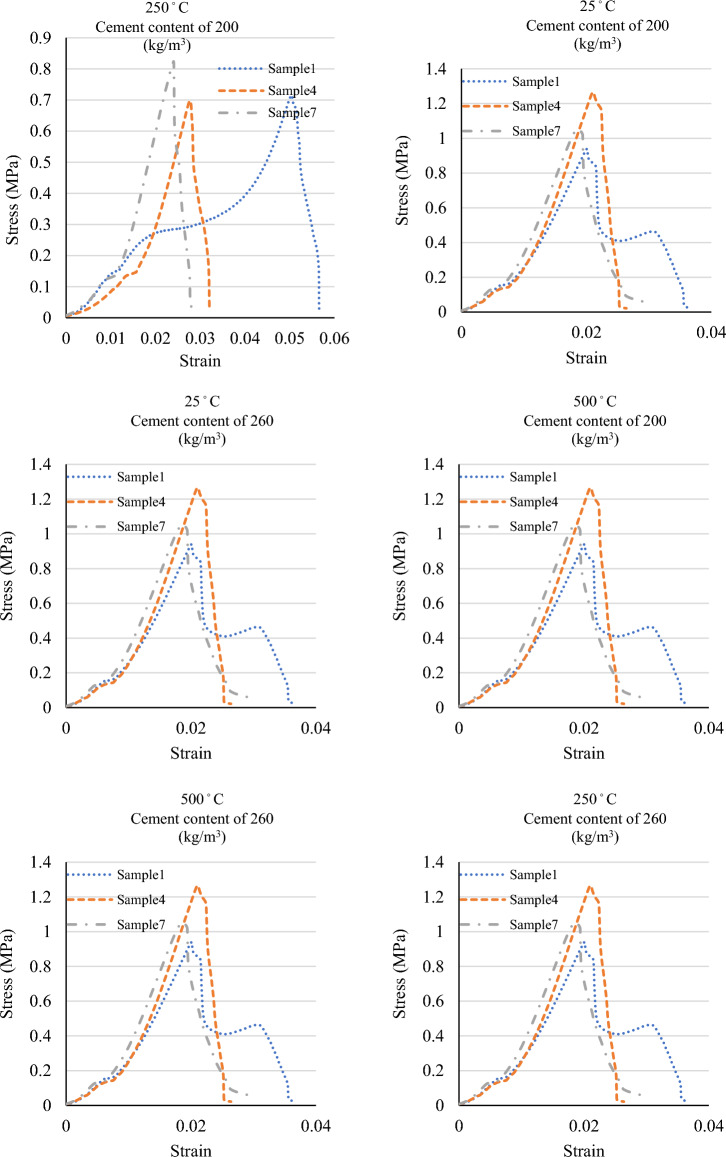

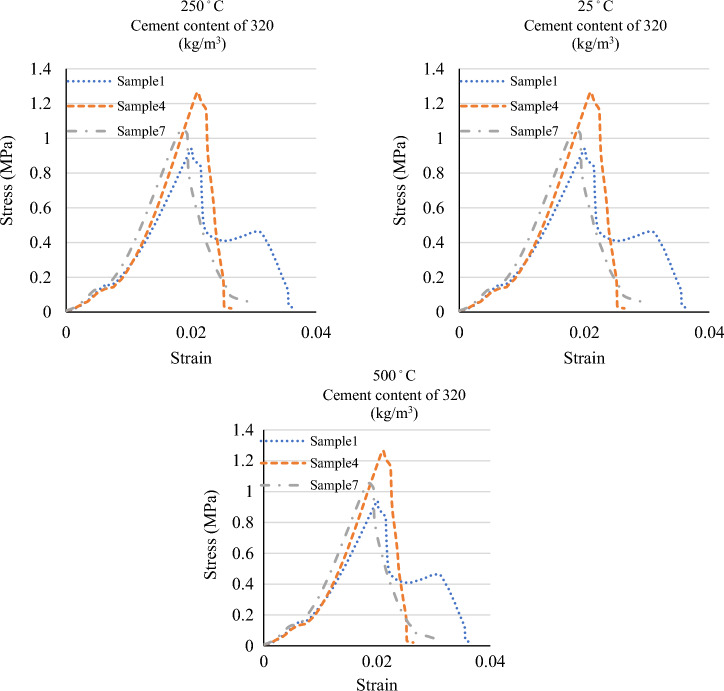
A comparison between the stress–strain graphs of the specimens at various temperatures and mix designs.

**Figure 12:**
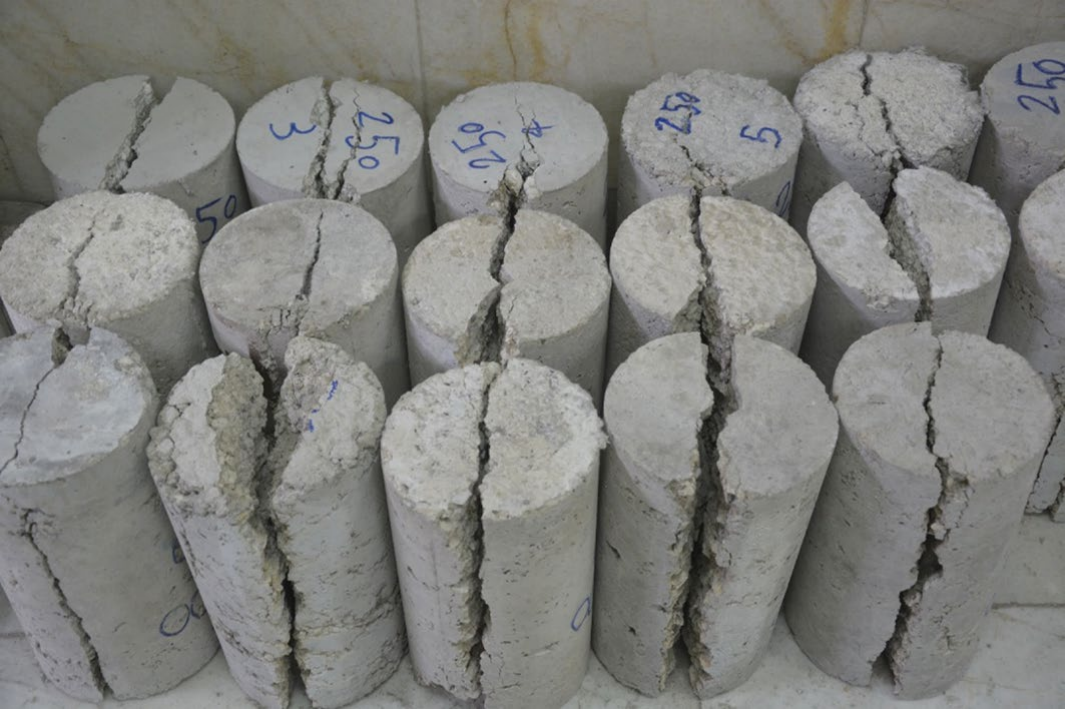
Cracks and fractures of samples under tensile test.

Figures 10 and 11, respectively, display the tensile strength and stress–strain curves obtained from the tensile test at fibre contents of 0.5%, 1.5%, and 2%. Also, The tensile strength was evaluated at the curing of 28 days of strength. Table 9 presents the percentage difference in the tensile strength of the specimens with 1.5% and 2% fibre from the tensile strength of those with 0.5% fibre. Figure 12 shows the cracks and fractures of samples under the tensile test**.**

**Table 9:** Percentage difference in the tensile strength relative to the specimen with 0.5% fiber.

Temperature of 500 °C			Temperature of 250 °C			Temperature of 25 °C			
Cement content of 320 (kg/m^3^)	Cement content of 260 (kg/m^3^)	Cement content of 200 (kg/m^3^)	Cement content of 320 (kg/m^3^)	Cement content of 260 (kg/m^3^)	Cement content of 200 (kg/m^3^)	Cement content of 320 (kg/m^3^)	Cement content of 260 (kg/m^3^)	Cement content of 200 (kg/m^3^)	Fiber content (%)
− 67	− 26	− 7	− 33	− 17	− 1	− 8	34	34	5.1
− 65	− 35	− 18	− 17	− 20	15	− 13	0	12	2

The tensile strength of specimens at 25 °C with a fibre content of 0.5%, 1.5%, and 2% was obtained to be, respectively, 0.94 MPa, 1.26 MPa, and 1.05 MPa for a cement content of 200 kg/m^3^; 1.01 MPa, 1.35 MPa, and 1.01 MPa for a cement content of 260 kg/m^3^; and 1.33 MPa, 1.23 MPa, and 1.26 MPa for a cement content of 320 kg/m^3^. In the specimens at 25 °C and with cement contents of 200 kg/m^3^ and 260 kg/m^3^, the highest tensile strength corresponds to the sample with 1.5% fibre. Also, the highest tensile strength in the specimens with a cement content of 320 kg/m^3^ belonged to the sample with 0.5% fibre.

The tensile strength of specimens at 250 °C with a fibre content of 0.5%, 1.5%, and 2% was determined to be, respectively, 0.71 MPa, 0.70 MPa, and 0.82 MPa for a cement content of 200 kg/m^3^; 1.07 MPa, 0.89 MPa, and 0.86 MPa for a cement content of 260 kg/m^3^; and 1.15 MPa, 0.77 MPa, and 0.96 MPa for a cement content of 320 kg/m^3^. In the specimens at 250 °C and with cement contents of 200 kg/m^3^, the highest tensile strength corresponds to the sample with 2% fibre. Moreover, the highest tensile strength in the specimens with cement contents of 260 kg/m^3^ and 320 kg/m^3^ belonged to the sample with 0.5% fibre.

The tensile strength of specimens at 500 °C with a fibre content of 0.5%, 1.5%, and 2% was obtained to be, respectively, 0.45 MPa, 0.42 MPa, and 0.37 MPa for a cement content of 200 kg/m^3^; 0.43 MPa, 0.32 MPa, and 0.38 MPa for a cement content of 260 kg/m^3^; and 0.79 MPa, 0.26 MPa, and 0.28 MPa for a cement content of 320 kg/m^3^. The highest tensile strength in specimens at a temperature of 500 °C with various cement contents corresponded to the sample with 0.5% fibre. Also, the tensile strength decreased with an increase in the fibre percentage.

The results showed that increased fibre content of the specimens subjected to temperatures of 250 °C and 500 °C decreased their tensile strength. The tensile strength of concrete was more sensitive to high temperatures than the compressive strength. Furthermore, PP fibers were more effective for compressive strength than tensile strength above 250 °C. Based on the test results, it can be concluded that adding 2 kg/m3 PP fibers can significantly promote the residual mechanical properties of concrete during heating^22^.

## Conclusion

This research examined the effect of temperature on the compressive and tensile strengths of concrete containing 0.5%, 1.5%, and 2% of polypropylene fibres and with cement contents of 200 kg/m^3^, 260 kg/m^3^, and 320 kg/m^3^. The main results obtained from the compressive and tensile tests are as follows:

1. The maximum improvement was noted to be 14.4 MPa for cement content of 320 kg/cm2 with 1.5% fiber at 28 days, and it shows a firm bonding characteristic among the PPFs and the matrix.
2. The behavior of concrete at high temperatures was evaluated, particularly the 1.5% fiber rate, which decreased strength loss more than other fiber rates. So, according to the experimental result, this type of concrete is suggested at normal tempreture.
3. The compressive strength decreased by 35% and 57% on average at 250 °C and 500 °C, respectively.
4. The tensile strength decreased by 26% and 65% on average at 250 °C and 500 °C, respectively.
5. An increase in the fibre content of the specimens at temperatures of 250 °C and 500 °C led to a decrease in compressive strength. Moreover, the compressive strength of the samples at a temperature of 25 °C increased for a fibre content of 1.5% and decreased for a fibre content of 2%.
6. An increase in the fibre content of the specimens subjected to temperatures of 250 °C and 500 °C caused a decrease in tensile strength.
7. The compressive strength increased with an increase in the cement content. Furthermore, no significant relationship was observed between cement content and tensile strength.
8. Mix Design 3, with a fibre content of 0.5% and a cement content of 320 kg/m3, exhibited higher compressive and tensile strengths at elevated temperatures compared to other designs.

## Data Availability

The datasets used and/or analyzed during the current study are available from the corresponding author upon reasonable request.
